# Complete mitochondrial genome sequence of the high altitude Brazilian treefrog *Pithecopus megacephalus* (Anura, Phyllomedusidae)

**DOI:** 10.1080/23802359.2019.1704184

**Published:** 2020-01-07

**Authors:** Nathália Gonçalves da Silva Lima, Anderson Oliveira do Carmo, Rafael Cerqueira Castro de Souza, Evanguedes Kalapothakis, Paula Cabral Eterovick

**Affiliations:** a Departamento de Biologia Geral, Instituto de Ciências Biológicas, Universidade Federal de Minas Gerais, Belo Horizonte, Brazil;; b Programa de Pós Graduação em Biologia de Vertebrados, Pontifícia Universidade Católica de Minas Gerais, Belo Horizonte, Brazil

**Keywords:** Mitochondrion, mtDNA, next-generation sequencing, Neobatrachia, treefrog

## Abstract

The first complete mitochondrial genome (mtDNA) for the family Phyllomedusidae (genus *Pithecopus*) is presented. It is a circular molecule with 17713 pb including 13 protein coding genes, 22 tRNA genes, two rRNA genes, and a control region (D-loop). *Pithecopus megacephalus* was close to the only other phyllomedusid whose complete mtDNA sequence is available, but had the cytb gene 147 pb smaller.

Several species of Phyllomedusidae are well known due to their complex peptidic skin secretions explored in biochemical research. Despite the great value of Phyllomedusidae for bioprospecting, the taxonomic and phyllogenetic relationships of its 60 taxa are still largely unresolved (Faivovich 2009).

*Pithecopus megacephalus* was described based on a single specimen and its type locality remains unknown (Miranda-Ribeiro 1926). It occurs in the Espinhaço Mountain Range, southeastern Brazil, and its distribution encompasses from Santana do Riacho municipality (Minas Gerais state) to Igaporã municipality (Bahia state; Brandão et al. 2012). It lives by streams in rocky meadows (Campos Rupestres) inserted in the Cerrado biome (Caramaschi 2006). Recent studies indicate high genetic variability for *P. megacephalus* (Ramos et al. 2018). Some of its skin peptides are similar to others with antimicrobial and antitumoral activities and are under investigation (Avelar-Júnior 2019).

A single mitogenome have been previously anotated for a phyllomedusid frog, *Callimedusa tomopterna* (JX564887), however its sequence is not complete at GenBank. Thus, we describe here the first complete mitogenome for Phyllomedusidae.

The mitogenome of *P. megacephalus* has 17713 pb. The GC content (41.6%) is considered high for Amphibia (mean of 37.4%; Lloyd et al. 2012), however not so different from other species of Hylidae such as *Hyla annectans* (39.4%), *H. chinensis* (40%), *H. tsinlingensis* (42.1%), and *Bokermannohyla alvarengai* (41.1%), as well as some Archaeobatrachia such as *Xenopus* (39.3% ± 3.6; Lloyd et al. 2012).

We extracted DNA from muscle of a young *P. megacephalus* following Herrmann and Frischauf (1987). We collected the frog at Catas Altas municipality (19°12′-19°20′S, 43°30′-43°40′W), Minas Gerais state, Brazil. We obtained a permit from Sisbio/ICMBio (48825-2) and deposited the tissue in the collection of the Universidade Federal de Minas Gerais (UFMG BDT AN1700001). We used Nextera DNA Sample Preparation Kit (Illumina, San Diego, CA, USA) for library construction following the manufacturer’s protocol. Sequencing was performed in MiSeq (Illumina) (MS), using MiSeq Reagent Kit v3 600 and paired-end strategy. *De novo* assembly was conducted in CLC Genomics Workbench 9.0 (Bio-Qiagen, Aarhus, Denmark) and final mtDNA sequence was annotated using MITOS (Bernt et al. 2013). We compared the complete annotated mtDNA of *P. megacephalus* with those of 16 anuran species available at GenBank using Mega 6 (Tamura et al. 2013), with the neighbour-joining algorithm (Figure 1).

**Figure 1.**
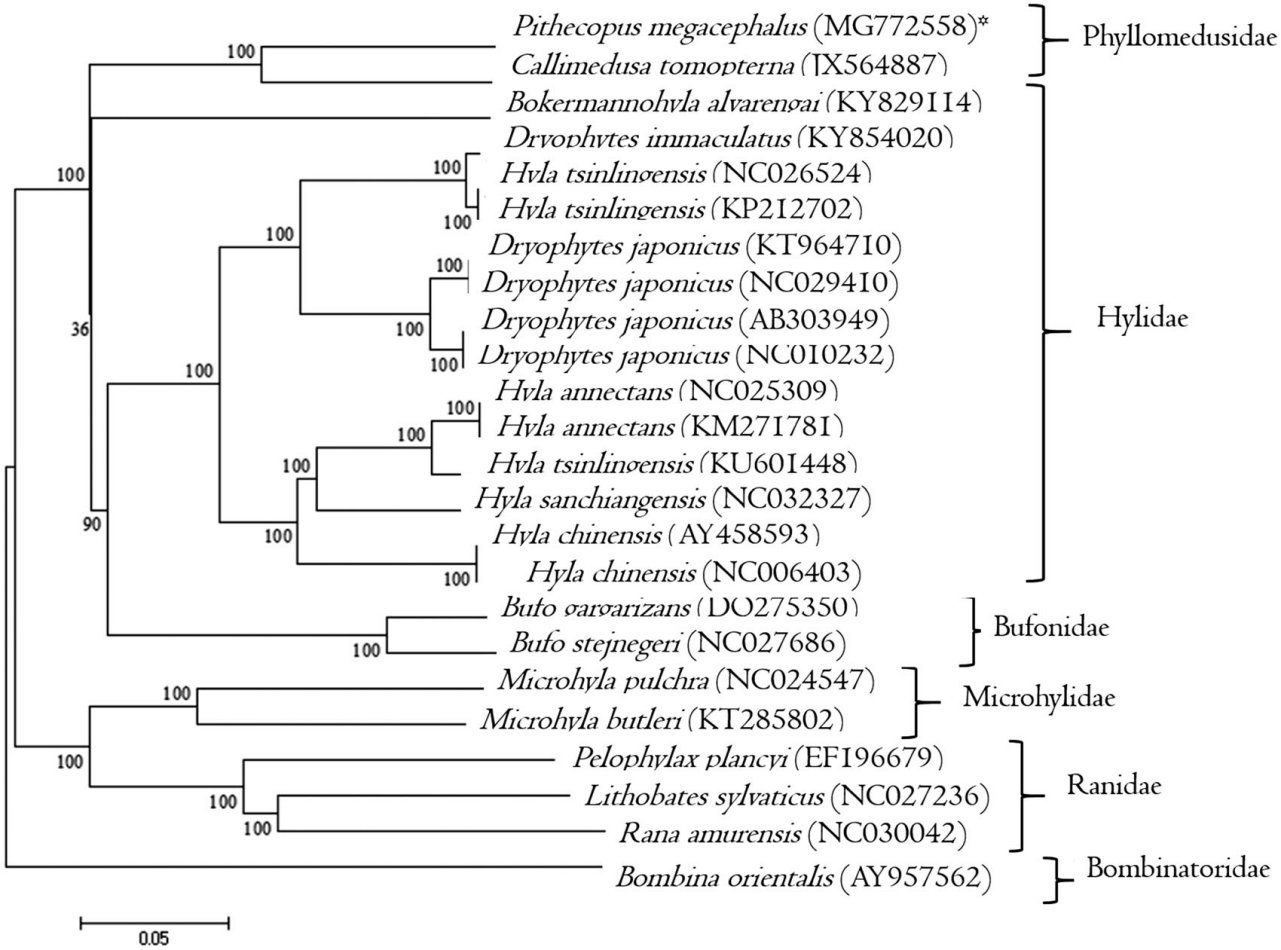
Neighbor-joining phyllogenetic tree of mtDNAs from anuran species: *Pithecopus megacephalus*, *Callimedusa tomopterna*, *Dryophytes japonicus*, *D. immaculatus*, *Hyla tsinlingensis, H. chinensis, H. sanchiangensis*, *H. annectans*, *Bokermannohyla alvarengai*, *Bufo gargarizans*, *B. stejnegeri*, *Bombina orientalis*, *Microhyla pulchra*, *M. butleri*, *Pelophylax plancyi*, *Lithobates sylvaticus,* and *Rana amurensis*. The phylogenetic tree was constructed under the Kimura-2 parameter model and consensus tree using 1000 bootstrap. Numbers indicate support of each clade.

The mtDNA of *P. megacephalus* (GenBank accession number MG772558) contains 2 rRNA genes (12 S rRNA and 16S rRNA), 22 tRNA genes, 13 protein coding genes (PCGs) and a control region (D-loop). It showed great conservatism in comparison with the partial mtDNA of *Callimedusa tomopterna* regarding gene content, size, and location, except for the cytb gene, which is 147 pb larger in *P. megacephalus.* Variations in gene order and content are rarely observed among related taxa (Alam et al. 2010). The cytb gene, however, is known to be very variable (Parson et al. 2000). Except from the genes nad5, nad6, cox1, and 6 tRNAs, the other genes in the mtDNA of *P. megacephalus* are coded in the heavy strand.
